# Do we need to reframe risk once again?

**DOI:** 10.4102/jamba.v15i1.1587

**Published:** 2023-11-22

**Authors:** Ian Christoplos, John Mitchell

**Affiliations:** 1 Danish Institute for International Studies, Strandgade, Copenhagen, Denmark; 2 British Red Cross, United Kingdom

**Keywords:** disaster mitigation, preparedness, discourse, reframing, jargon

## Abstract

Twenty years ago we wrote an article entitled ‘Re-framing risk: The changing context of disaster mitigation and preparedness’. We sought to summarise the changes that were underway at the time in the discourse on disaster risk. At the time the article was seen as rather provocative as it sought to summarise the way that new perspectives were emerging in how we perceived risk. Ben Wisner nudged us to reflect on what happened to that reframing and whether it may be time to re-frame things once again. This reflection has led to several streams of thought.

## Changes in jargon and scope – Content, not so much

Returning to the original re-framing article, the first thought that comes to mind is that the jargon is rather more dated than the content. Most notably, even if the number of programmes and experts working with disaster risk have multiplied considerably and although resources have grown as well, the field still suffers from tendencies to fall between the cracks of different policies and silos of development and humanitarian practice. This is despite the growing awareness that crises are converging. Disaster risk reduction (DRR) (which we referred to back then as disaster mitigation and preparedness) is still not quite humanitarian action or development. Indeed, now the topic needs to contend with expectations that it should be integrated with climate change adaptation, peacebuilding, pandemic response, among others. Furthermore, it is now expected that risk should be managed through ‘Grand Bargains’ such as that proclaimed at the World Humanitarian Summit in 2016 among actors across the humanitarian and development communities, ‘New Ways of Working’ (one of various slogans that have come and gone calling for a different relationship between the international community and host governments) and reinforced commitments to ‘leave no one behind’, the central call from the Sustainable Development Goals. Expectations have grown, but clarity remains elusive.

## Changing gaps in the discourse

Looking back more than two decades, the main difference now is thus that the gaps that characterise the framing of disaster risk reduction (or nexuses as they are more optimistically referred to these days) have multiplied. The most obvious gap is in managing the linkages to climate change adaptation. Since we wrote the article, ‘Re-framing risk: The changing context of disaster mitigation and preparedness’ (Christoplos, Mitchell & Liljelund 2001), countless analyses of this relationship have been undertaken and some of the programming is far more integrated. However, the status quo remains rather mixed. It has become standard practice to claim that programmes reflect recognition of how a significant proportion of climate change adaptation needs to be focused on disaster risk, but there are many missed opportunities, some of which relate to the dysfunctions we observed 20 years ago. Climate policy people think with longer time horizons, and those managing disaster risk tend to focus on much shorter timeframes. Sub-national authorities and civil society tend to be overwhelmed by the scope and time frame of climate change adaptation policies and plans and prefer to stick to more concrete disaster risk management efforts (Christoplos et al. 2017). There is much more of a conversation underway now about the linkages between disaster risk and climate change, but the communities have not fully merged.

In our original article on re-framing we noticed how ‘complex political emergencies’ had then taken the limelight. The complex political emergency discourse (e.g. Cliffe & Luckham 1999) has in recent years morphed into a concern for dealing with the humanitarian-development-peace nexus based on reinforced political economy analyses (sort of a complex political version of what was then referred to as linking relief, rehabilitation and development). Disaster risks are sometimes factored into this nexus, but certainly not always. The concern for disasters within the nexus is most apparent where these are seen to trigger conflict and migration, but there has been notably little actual integration of risk reduction response into thinking around ‘the nexus’. The technical people working with disaster risk rarely engage with the peacebuilders. Natural hazard early warning systems remain separate from conflict early warning. Efforts to ‘crunch the numbers’ across these two measurement systems and realms of response have not been very successful. In sum, there are seemingly obvious political complexities around bringing together information that remain insufficiently confronted (Maxwell & Hailey 2020).

## Old ruts remain under new labels

In our article we drew attention to the problems associated with technocratic ruts and projectisation that stand in the way of longer-term efforts reflecting the multiple ways that people are trying to manage risk. The projectisation tendencies may have become worse because of even shorter-term political and donor agendas, including the lure of being able to show short-term climate change results, as well as stopping migration flows, within election cycles. Despite acknowledgement that addressing complex risk requires long timeframes, 6 month humanitarian projects are still expected to demonstrate risk reduction ‘outcomes’. It has also perhaps become even easier to remain in a technical bubble when we communicate by Zoom and are more rarely confronted, face-to-face, with the shortcomings of our technical models, absurd implementation timeframes, or (most of all) the situation of the disaster affected people who have to deal with these models. Those designing and deciding on financing of projects are more distant than ever from seeing what risk looks like.

New jargon has emerged that is expected to signal a commitment to deeper reflection. A rapidly emerging agenda now is that of anticipatory action (see https://www.unocha.org/our-work/humanitarian-financing/anticipatory-action), being promoted by the United Nations Office for the Coordination of Humanitarian Affairs (OCHA), the World Food Programme (WFP) and the Food and Agricultural Organisation (FAO), or early warning and/or early action and/or response as it is sometimes still referred to. Particularly when these efforts include elements of ‘foresight’ and the struggle to achieve ‘resilience’, this may lead to conscious efforts to do what we did in the past, that is, mitigate and prepare for disasters, even though that sounds rather old-fashioned. These agencies have recognised that anticipation should be about addressing risk before disasters strike. Hardly a new discovery.

## Still struggling with actors’ roles

Another area we took up in the article was the changing roles and relationships among the actors involved. Even though disaster risk is rarely high on the agenda in these discussions, the commitments from the World Humanitarian Summit in 2016, the Grand Bargain, the New Way of Working and particularly the repeated attempts to act on calls for localisation are all new iterations of these concerns about the need for changes in the roles of actors in risk. It would appear that these more recent commitments are somewhat more concerted, even if the outcomes still leave much to be desired. Oddly enough, the discourse on disaster risk is perhaps ill-fitted to a discourse about how we need to now start localising. It has long been assumed that national authorities and local communities already are the main actors. Disaster risk reduction is assumed to be pre-localised and does not need an extra push, despite the fact that readiness to actually hand over DRR resources to the control of these local actors remains limited.

## A modest reframing makes sense, but the key questions remain about how to move ahead

In our article we stated that the ‘discourse has begun to shift from a focus on *which* technical choice is most appropriate to a concentration on the political process that determines *how* these choices are made’. In retrospect, today there is greater recognition that this is what we need to focus on, but it is not certain whether we have figured out how to do it. In some respects, there is a greater concern than ever about how to move forward as the volume of calls to ‘do something’ about converging crises has reached feverish levels. When overwhelmed by political complexity, many may retreat back to technical ‘fixes’. This is reinforced as many key actors are more distant than ever from being able to see what disaster risk means for vulnerable people and for the local and global institutions that need to act if we are to overcome the inertia that has plagued risk reduction.
