# Corrigendum: Do we need to reframe risk once again?

**DOI:** 10.4102/jamba.v16i1.1727

**Published:** 2024-07-17

**Authors:** Ian Christoplos, John Mitchell

**Affiliations:** 1 Glemminge Development Research, Glemmingebro, Sweden; 2 Active Learning Network for Accountability and Learning in Humanitarian Action (ALNAP), London, United Kingdom

In the original published article, Christoplos, I. & Mitchell, J., 2023, ‘Do we need to reframe risk once again?’, *Jàmbá: Journal of Disaster Risk Studies* 15(1), a1587. https://doi.org/10.4102/jamba.v15i1.1587. There was an error in the affiliation of author 1 and author 2.

Instead of ‘Danish Institute for International Studies, Strandgade, Copenhagen’ for author 1, Ian Christoplos, it should be: ‘Glemminge Development Research, Glemmingebro, Sweden’.

Instead of ‘British Red Cross, United Kingdom’ for author 2, John Mitchell, it should be: ‘Active Learning Network for Accountability and Learning in Humanitarian Action (ALNAP), London, United Kingdom’.

The authors apologise for this error. The correction does not change the study’s findings its significance or overall interpretation of its results or the scientific conclusions of the article in any way.

